# Efficacy of a single-file reciprocating protocol vs. a multi-file rotary protocol on the removal of gutta-percha and ceraSeal sealer from oval canals: a volumetric and active instrumentation time analysis

**DOI:** 10.3389/fdmed.2026.1926115

**Published:** 2026-10-14

**Authors:** Ahmed Zorek, Abeer H. Mahran, Mohammed Nabeel, Tarek Elsewify

**Affiliations:** 1 Endodontic Department, Misr International University, Cairo, Egypt; 2 Endodontic Department, Ain Shams University, Cairo, Egypt; 3 Restorative Dental Science Department, Gulf Medical University, Ajman, United Arab Emirates

**Keywords:** bioceramic sealer, cone-beam computed tomography, continuous rotation, oval root canals, reciprocation, retreatment

## Abstract

**Objective:**

To evaluate and compare the overall efficacy and time efficiency of two complete manufacturer-recommended retreatment protocols, a single-file reciprocating protocol, Reciproc (RC), vs. a multi-file continuous rotary protocol, ProTaper Universal Retreatment (PR), in removing gutta-percha and CeraSeal sealer from oval-shaped root canals.

**Methodology:**

Thirty-six single-rooted human mandibular premolars with single oval canals were selected, instrumented, and obturated with a matching cone gutta-percha and CeraSeal sealer. Initial filling volumes were evaluated via CBCT. The samples were randomly divided into two experimental groups (*n* = 18) according to the file used for retreatment. The net active instrumentation time was recorded. Post-retreatment CBCT scans were performed to measure the volume and percentage of remaining filling materials. Data were statistically analyzed at a significance level of *p* ≤ 0.05.

**Results:**

Both systems significantly reduced the filling volume (*p* < 0.0001). No significant difference was found in the initial filling volumes between groups (*p* = 0.616). However, the RC group demonstrated a significantly lower mean volume (0.474 ± 0.313 mm^3^) and percentage (5.52 ± 1.97%) of remaining filling material compared to the PR group (1.735 ± 0.753 mm^3^ and 20.72 ± 2.93%, respectively; *p* < 0.0001). Furthermore, the RC group required significantly less active instrumentation time (48.69 ± 4.98 s) than the PR group (68.49 ± 5.43 s, *p* < 0.0001).

**Conclusion:**

Within the limitations of this *in vitro* study, neither mechanical retreatment protocol completely removed CeraSeal sealer and gutta-percha from oval root canals. However, the single-file RC protocol was significantly more effective and time-efficient than the multi-file PR protocol. The superior performance of the RC protocol reflects the combined effect of its matched apical size (size 40 vs. size 20), single-file sequence, cross-sectional design, and reciprocating kinematics, rather than kinematic motion alone.

## Introduction

One of the objectives of non-surgical endodontic retreatment is the maximum possible removal of previous filling materials from the root canal space. Thorough removal is critical to expose persistent intracanal microorganisms, allow adequate contact of irrigating solutions with the dentinal walls, and facilitate effective disinfection (1).

Achieving complete cleanliness is particularly complex in oval-shaped root canals, such as those frequently present in human mandibular premolars. Rotary endodontic files tend to prepare a circular standardized canal, often leaving uninstrumented buccal and lingual recess extensions where filling remnants remain sheltered (2). This clinical challenge is further compounded by the introduction of modern calcium silicate-based sealers. Calcium silicate-based sealers, such as CeraSeal (Meta Biomed, Cheongju, South Korea), exhibit superb physical and biological attributes, including high alkalinity, negligible setting shrinkage, and deep dentinal tubule penetration. However, their chemical bond to root dentin and resistance to chemical solvents make them highly challenging to remove mechanically during retreatment (3–5).

To improve removal efficiency, various nickel-titanium systems with different file sequences and kinematic behaviors have been utilized (6). Multi-file rotary systems, such as ProTaper Universal Retreatment (PR), utilize three sequentially designed files (D1, D2, and D3) to progressively eliminate materials from the coronal, middle, and apical thirds of the root canal under continuous rotation (7, 8). Alternatively, single-file reciprocating systems, such as Reciproc (RC), operate using unequal counter-clockwise and clockwise cycles. This mechanical approach is designed to minimize torsional stress, enhance file flexibility, and allow a single robust instrument to rapidly advance through obturation masses (9).

Evaluating residual filling material requires non-destructive three-dimensional assessment. While conventional radiography produces overlapping two-dimensional projections, cone-beam computed tomography (CBCT) provides quantitative volumetric assessment at clinical CBCT resolution (10). Utilizing clinical CBCT parameters enables the evaluation of retreatment outcomes under diagnostic imaging conditions representative of contemporary endodontic practice.

Consequently, this *in vitro* study aimed to evaluate and compare the overall efficacy of two complete manufacturer-recommended retreatment protocols, the single-file reciprocating system RC vs. the multi-file continuous rotary system PR, on the volume and percentage of remaining calcium silicate-based sealer/gutta-percha inside oval root canals using three-dimensional CBCT analysis, as well as the absolute operating time required for material removal. The null hypothesis tested was that there would be no significant difference between the two systems regarding volumetric cleaning efficiency or procedural speed.

## Mehtodolgy

### Study design and ethical approval

This experimental, parallel, randomized, comparative *in vitro* study was approved by the institutional ethics committee; approval number: MIU-IRB-2223-184.

### Sample size calculation

A power analysis was designed to have adequate power to apply a two-sided statistical test of the null hypothesis. By adopting an alpha level of (0.05), a beta of (0.2), i.e., power = 80%, and an effect size (d) of (0.963) calculated based upon the results of Aly et al. (11), the predicted sample size (n) was a total of (36) teeth (18 per group). Sample size calculation was performed using G*Power version 3.1.9.7.

### Sample selection and root canal preparation

Thirty-six freshly extracted human mandibular premolars, each possessing a single root canal, with mature roots, fully formed apices, and single oval-shaped root canals were selected. Preoperative high-resolution digital radiographs were taken from buccolingual and mesiodistal angles to confirm a single canal without internal calcifications or previous treatments. The buccolingual diameter was at least double the mesiodistal diameter. The anatomic crowns were sectioned at the cementoenamel junction using a water-cooled diamond disc to standardize a uniform root length of 16 mm.

Canal patency was established by inserting a size 10 K-file into the canal until the tip emerged at the apical foramen. The working length was established at 15 mm (1 mm short of the apical foramen). The root canals were mechanically instrumented using the ProTaper Next system (Dentsply Sirona, Ballaigues, Switzerland) up to file X4 (size 40,.06 taper) at a speed of 300 rpm and torque of 2.0 N·cm. Chemical irrigation was performed between each file change using 5 mL of 2.5% sodium hypochlorite. Final rinse sequences included 2 mL of 17% ethylenediaminetetraacetic acid for one minute to remove the smear layer, followed by a final flush with 5 mL of distilled water. The root canals were partially dried using a single sterile matching paper point.

### Root canal obturation

The prepared root canals were obturated using the matching-cone technique. CeraSeal bioceramic sealer (Meta Biomed, Cheongju, Korea) was injected into the coronal third of the canal using the manufacturer's intracanal tip. A standardized matching gutta-percha master cone (size 40,.06 taper) was coated with a thin layer of sealer and slowly inserted to the full working length. Excess gutta-percha was sheared off at the root orifice using a heated plugger, and light vertical condensation was applied. Postoperative radiographs confirmed dense, well-adapted root canal fillings without voids. The specimens were incubated at 37 °C and 100% relative humidity for 14 days to ensure the calcium silicate-based sealer set completely.

### Pre-retreatment volumetric imaging (CBCT)

All specimens were positioned in a customized jaw jig and scanned using a CBCT machine (Vatech Pax-i3D, Vatech, Yongin, Korea) operating at 85 kV, 10 mA, with a voxel size of 0.12 mm. The resulting images were exported in DICOM format and imported into OsiriX medical imaging software (Pixmeo, Geneva, Switzerland). Segmentation of the root canal filling material was performed manually on each axial slice using the polygon selection tool in OsiriX software. Because CBCT gray-scale values are influenced by acquisition parameters and do not represent standardized Hounsfield Units, a fixed threshold value was not applied. Instead, the filling material was identified based on its radiopaque boundaries relative to the surrounding dentin and canal space. All specimens were assessed using identical image display settings (window level and window width) throughout the evaluation process to ensure consistency in material identification. The third author used the polygon manual selection tool to trace and segment the high-density root filling core (gutta-percha and CeraSeal sealer combined) slice-by-slice across the entire length of the canal. The software compiled these segments into a 3D volume reconstruction to calculate the pre-retreatment volume of filling material (V_initial_ in mm^3^), Figure 1.

**Figure 1:**
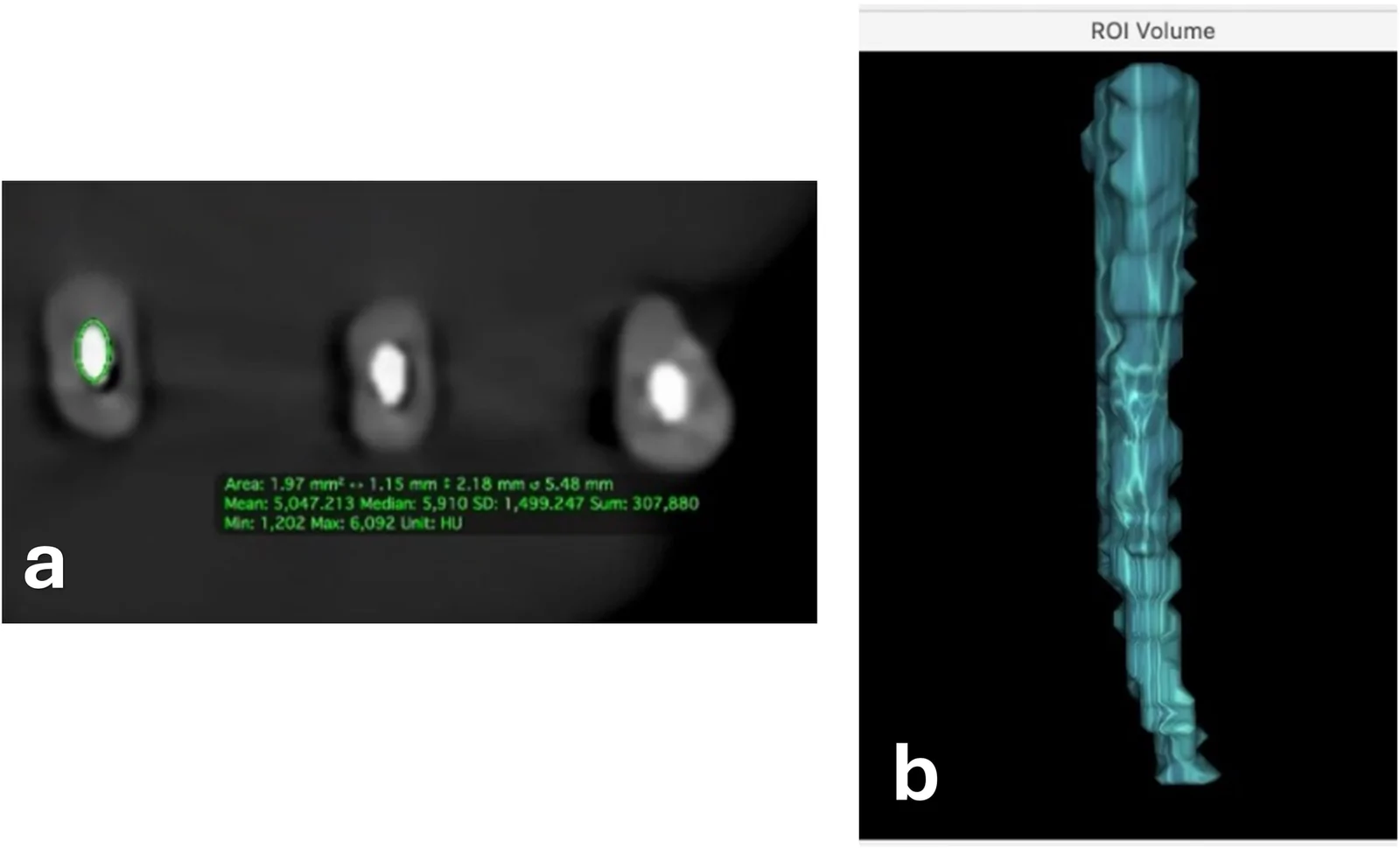
Volumetric evaluation, **(a)** using the polygon tool to manually outline the root canal filling materials in each slice of the axial cut; **(b)** osirix software displaying the 3D model of the segmented filling material core and the volume of the root canal filling material.

### Randomization and group allocation

The 36 prepared specimens were randomly assigned in equal proportions (*n* = 18) to the two experimental groups using a computer-generated random number sequence (https://www.random.org). Randomization and allocation concealment were managed by an independent researcher who assigned randomized numerical identification codes to the specimens prior to retreatment. The key linking sample codes to group assignments was kept sealed until all CBCT volumetric segmentations and statistical evaluations were completed.

### Retreatment protocols

Group PR: Material removal was accomplished using PR (Dentsply Sirona, Ballaigues, Switzerland) driven by an electric motor at 500 rpm (torque 3.0 N·cm) for D1 and 400 rpm (torque 2.0 N·cm) for D2 and D3. File D1 (30/.09) was applied to the coronal third; file D2 (25/.08) was advanced into the middle third; and file D3 (20/.07) was used to reach the full working length. The files were used with a gentle brushing action against the canal walls.

Group RC: Material removal was performed using a single RC R40 instrument, size 40, .06 taper (VDW, Munich, Germany) operated via an electric motor set to the pre-programmed “RECIPROC ALL” mode. The R40 file was inserted into the canal orifice using a gentle, slow in-and-out pecking motion with an amplitude of no more than 3 mm. After every three pecks, the file was withdrawn and cleaned of debris. The preparation was advanced until the tool reached the full working length.

No chemical solvents were utilized in either group. To maintain standardization, the total irrigation volume was fixed at 6 mL of 2.5% sodium hypochlorite (NaOCl) per specimen in both groups. In Group PR, irrigation was delivered after each of the three instruments (D1, D2, and D3) using 2 mL of 2.5% NaOCl per change. In Group RC, reaching the full working length required three pecking sequences; irrigation was performed after each 3-peck sequence using 2 mL of 2.5% NaOCl. Following active retreatment, all specimens in both groups received an identical final rinse protocol consisting of 2 mL of 17% EDTA for 1 min followed by 2 mL of distilled water, ensuring a completely standardized overall irrigant volume (10 mL total per tooth) across all samples.

In both experimental groups, active mechanical instrumentation was governed by objective operational endpoints: retreatment was completed when the designated final instrument (Reciproc R40 or ProTaper D3) successfully reached full working length (15 mm) and completed a standardized irrigation sequence. Supplementary clinical confirmation was performed under a dental operating microscope to verify clear irrigant effluent, absence of visible obturation debris on the file flutes, and smooth canal wall tactile feedback. The first author did all the procedures using the dental operating microscope.

### Active instrumentation time measurement

The net active instrumentation time required for filling material removal was recorded in seconds using a digital stopwatch. Irrigation delivery, instrument changes, flute cleaning, and visual inspection procedures were excluded from the measurement to isolate the mechanical cutting efficiency of the tested instruments.

### Post-retreatment volumetric imaging

Following retreatment, the specimens were scanned again using the same CBCT parameters. Rather than employing qualitative visual scoring scales, objective continuous 3D volumetric criteria (mm^3^) were utilized to evaluate pre- and post-operative differences within the root canal lumen. The residual filling material volume (V_residual_ in mm^3^) was computed using the same slice-by-slice manual segmentation method in OsiriX software. The percentage of remaining filling material (%R_filling_) was calculated using the following formula (12, 13):

$$\text{\%}R_{\mathrm{filling}}=\frac{V_{\mathrm{residual}}}{V_{\mathrm{initial}}}\times 100$$

Prior to the experimental measurements, the evaluator was calibrated using a series of representative CBCT datasets not included in the final analysis to standardize identification and delineation of the filling material boundaries. To ensure blinding during outcome assessment, all CBCT DICOM datasets were anonymized and assigned randomized numerical codes by an independent researcher. The evaluator (third author) who conducted the manual polygon segmentation and volumetric calculations (V_initial_ and Vresidual in mm^3^) using OsiriX medical imaging software was completely blinded to the experimental group assignments. Group codes were revealed only after completion of all volumetric measurements.

To ensure measurement reproducibility using the manual polygon tool, intra-examiner reliability was evaluated. The third author re-analyzed a random 20% subset of the pre- and post-retreatment CBCT scans (*n* = 8) two weeks following the initial segmentation, while blinded to the previous data. Reliability was quantified using the Intraclass Correlation Coefficient (ICC) based on a two-way mixed-effects model.

### Statistical analysis

Data were analyzed using IBM SPSS Statistics Version 26 (IBM Corp., Armonk, NY, USA). Normality of continuous data distribution was assessed using Kolmogorov–Smirnov and Shapiro–Wilk tests (*p* > 0.05). Homogeneity of variances between groups was evaluated using Levene's test. Pre-retreatment and post-retreatment volumes within each system were analyzed using paired *t*-tests. Intergroup comparisons regarding residual filling volume, percentage of remaining filling material, and procedural time were performed using Welch's *t*-test to account for unequal sample variances, alongside the non-parametric Mann–Whitney *U*-test as a sensitivity check. To control for family-wise type I error rate across multiple primary outcomes, the Bonferroni correction was applied, adjusting the significance threshold to alpha = 0.0167 (0.05/3). Furthermore, the relationship between net instrumentation time and percentage of residual filling material was evaluated using Pearson (r) and Spearman (*ρ*) correlation coefficients. Overall statistical significance was set at *p* < 0.05.

## Results

### Volumetric CBCT evaluation

The intra-examiner reliability testing for manual slice-by-slice volumetric segmentation demonstrated excellent agreement, yielding an ICC of 0.96 (95% CI: 0.91–0.98).

The baseline volumetric assessment verified that the root canal filling cores were uniformly distributed between the experimental cohorts before the retreatment protocols, with no statistically significant difference in baseline obturation volumes between the two groups (*P* = 0.616), confirming structural uniformity across samples before intervention.

Intragroup statistical evaluations showed that both file systems generated a highly significant reduction in obturation material volume compared to their respective baseline volumes (*P* < 0.0001). Neither mechanical instrument protocol succeeded in achieving complete removal of the CeraSeal bioceramic sealer and gutta-percha from the oval canal spaces.

Levene's test revealed significant heterogeneity of variance between experimental groups for residual filling volume (*p* = 0.004). Using Welch's *t*-test, intergroup comparisons demonstrated that the RC group left a significantly lower mean residual volume (0.474 ± 0.313 mm^3^) and percentage of residual filling material (5.52 ± 1.97%) compared to the PR group [1.735 ± 0.753 mm^3^ and 20.72 ± 2.93%, respectively; Welch's *t*(22.7) = 6.56, *p* < 0.0001 for volume; Welch's *t*(29.8) = 18.27, *p* < 0.0001 for percentage]. The non-parametric Mann–Whitney *U*-test confirmed these findings (*U* = 0.00, *p* < 0.0001). All intergroup differences remained highly significant after applying the Bonferroni correction threshold (alpha adjusted = 0.0167).

### The net active instrumentation time

The RC was significantly faster and required a mean active instrumentation time of 48.69 ± 4.98 s compared to the PR 68.49 ± 5.43 s (*P* < 0.0001), Table 1.

**Table 1:** Volumetric and chronological comparisons between the RC and PR during the removal of gutta-percha and bioceramic sealer from oval root canals.

Variable	Reciproc (*n* = 18)	ProTaper universal retreatment (*n* = 18)	Welch's *t*-statistic (df)	*P*-value
Initial volume mean ± SD in mm^3^	8.58 ± 1.12	8.38 ± 1.25	*t* = 0.51 (33.6)	0.616 ns
Residual volume mean ± SD in mm^3^	0.474 ± 0.313	1.735 ± 0.753	*t* = 6.56 (22.7)	<0.0001*
Percentage of remaining volume mean ± SD	5.52 ± 1.97	20.72 ± 2.93	*t* = 18.27 (29.8)	<0.0001*
Active Instrumentation Time mean ± SD in seconds	48.69 ± 4.98	68.49 ± 5.43	t = 11.38 (33.7)	<0.0001*

SD, Standard Deviation; df: Welch-Satterthwaite adjusted degrees of freedom.

* Statistically significant difference between groups (*p* < 0.0167, applying Bonferroni correction for multiple comparisons). Intergroup comparisons were evaluated using Welch's *t*-test for unequal variances and verified using the non-parametric Mann–Whitney *U*-test (*P* < 0.0001 for all significant outcomes). ns: Not statistically significant (*P* > 0.0167).

### Correlation between active instrumentation time and cleaning efficacy

Correlation analysis across the total sample demonstrated a strong positive correlation between net active instrumentation time and the percentage of residual filling material (*r* = 0.887, *p* < 0.0001; *ρ* = 0.862, *p* < 0.0001). However, within-group correlation analyses revealed no statistically significant relationship between operating time and residual material percentage in either the RC group (*r* = 0.124, *p* = 0.623) or the PR group (*r* = −0.089, *p* = 0.725). This indicates that the overall correlation was driven by system-level differences between the two protocols rather than individual operator speed variations within a group.

## Discussion

Complete removal of the root canal filling material is challenging due to anatomical variations like oval configurations and the adhesive nature of bioceramic sealers. This study evaluated the efficiency and procedural speed of single-file reciprocating and multi-file rotary systems when removing gutta-percha and CeraSeal bioceramic sealer from oval root canals.

Human mandibular premolars were selected for this study because they frequently present a single root canal with an oval cross-section. Rotary nickel-titanium instruments inherently prepare a standardized circular path inside the root canal. Consequently, in an oval configuration, these instruments often fail to contact the anatomical recess extensions located at the buccal and lingual aspects, leaving uninstrumented zones where filling material remnants remain shielded.

To simulate a demanding clinical retreatment scenario within these anatomical challenges, the canals were obturated using a single-cone technique combined with CeraSeal bioceramic sealer. CeraSeal is a premixed calcium silicate-based material that undergoes a hydration reaction with moisture inside the dentinal tubules, yielding calcium silicate hydrates and calcium hydroxide. This chemical process generates a hard, insoluble matrix that establishes a tight micromechanical bond with the root dentin (3, 14–16). While this enhances the primary three-dimensional seal, it renders the material highly resistant to mechanical removal and conventional chemical endodontic solvents. Therefore, to evaluate the pure mechanical cutting and removal efficiency of the tested file systems, no chemical solvents were utilized during the retreatment protocols.

To assess residual filling material within the canal lumen, CBCT was deliberately selected as a clinically available three-dimensional imaging modality. Assessing retreatment outcomes under imaging conditions encountered in daily endodontic practice enhances the translational clinical relevance of these findings, as CBCT represents the primary 3D diagnostic tool accessible to clinicians for post-treatment evaluation. CBCT scans combined with slice-by-slice manual segmentation via OsiriX software allowed for the precise quantification of initial and post-retreatment filling volumes (mm^3^). While conventional two-dimensional radiography suffers from anatomical superimposition and geometric distortion, CBCT provides a longitudinal, non-destructive volumetric assessment across the entire root canal space without requiring tooth destruction or sectioning. Furthermore, because clinical CBCT represents the exact diagnostic imaging tool available in daily endodontic practice, assessing retreatment protocols under standard clinical CBCT resolution enhances the translational clinical relevance of these findings (17, 18).

Based on our results, the null hypothesis was rejected, as significant differences were observed between the two systems for both residual filling percentages and net operating times (*P* < 0.0001). The volumetric findings of this study revealed that neither the single-file reciprocating system nor the multi-file rotary system could completely remove the bioceramic sealer and gutta-percha from oval root canals, which is consistent with existing endodontic literature. However, the RC demonstrated a significantly higher cleaning efficacy, leaving a significantly lower percentage of remaining filling volume compared to the PR.

The superior cleaning capacity of the RC protocol can be attributed to the synergistic combination of its instrument dimensions, cross-sectional design, file sequence, and kinematic motion. The RC R40 instrument was sized to match the master apical preparation diameter (size 40, .06 taper), enabling direct mechanical contact across a larger surface area of the canal core compared to the smaller terminal diameter of the PR D3 file (size 20, .07 taper). Furthermore, the R40 features an S-shaped cross-section with sharp two-point cutting edges and progressive flutes that provide large chip spaces for coronal debris displacement. These structural design features, combined with unequal reciprocating kinematics (150° counter-clockwise cutting/30° clockwise release) that prevent file binding in hardened bioceramic mass, allowed the single-file RC protocol to remove filling material more effectively than the multi-step PR protocol (18–20).

These results are in agreement with Bago et al. (21) and Rios et al. (22), who reported that reciprocating kinematics left significantly less residual filling material in complex canal anatomies compared to continuous rotation systems.

However, in agreement with micro-CT evaluations by Orhan et al. (9) and Eid et al. (6) single-file reciprocating kinematics demonstrated significantly higher volume reduction than multi-file continuous rotary systems. Micro-CT cross-sectional analyses show that the reciprocating motion, combined with S-shaped file geometry, facilitates better central displacement of stubborn obturation masses without burnishing softened debris into anatomical irregularities, a common limitation observed under continuous rotation. Nevertheless, because micro-CT studies confirm the presence of microscopic sealer remnants post-instrumentation, mechanical retreatment must be supplemented with active irrigation modalities to clean areas untouched by engine-driven instruments.

In contrast, the PR system operates under continuous rotation and relies on a multi-file sequence (D1, D2, and D3). Although these files feature a convex triangular cross-section that provides high torsional strength, their smaller blade chip spaces can limit debris clearance when cutting dense materials. Under continuous rotation, the friction generated can soften the gutta-percha, causing the rotary blades to burnish and pack a mixture of softened gutta-percha and set bioceramic particles into the uninstrumented buccal and lingual extensions of the oval root canals rather than extracting them. This mechanical limitation explains the significantly higher percentage of residual filling material observed in the PR group (9).

Regarding time efficiency, the RC protocol required significantly less active instrumentation time than the PR protocol. A methodological choice in this study was to report net active instrumentation time rather than overall chairside procedural time, thereby isolating mechanical cutting efficiency from operator-dependent variables such as irrigation speed or instrument swapping. It should be noted that in clinical practice, total chairside time for the multi-file PR protocol would be even longer when accounting for instrument changes (D1 to D2 to D3) and repeated flute cleanings. Thus, single-file reciprocating protocols optimize overall clinical workflow efficiency by eliminating non-active instrument handling overhead.

This difference is primarily driven by protocol complexity and file sequence. The PR system requires the sequential use of three separate instruments to progress safely from the coronal (D1) to the middle (D2) and apical (D3) thirds of the root canal. This multi-step progression naturally prolongs the active instrumentation time (23). Conversely, the RC system utilizes a single, robust instrument (R40) to reach the full working length using a standardized pecking motion, which drastically minimizes instrument manipulation and handling time (18).

This finding aligns with Souza et al. (24), who concluded that single-file reciprocating kinematics optimize clinical workflows by removing root fillings significantly faster than conventional multi-file rotary sequences.

The reported time values represent active instrumentation time rather than total clinical retreatment time. Therefore, the findings should be interpreted as a comparison of the mechanical removal efficiency of the tested systems under standardized experimental conditions. Actual chairside retreatment time would also include irrigation procedures, instrument exchanges, cleaning, and clinical assessment steps.

Our volumetric findings show that both tested files failed to totally remove the filling material. This aligns with Buyuksungur et al. (25) and Colombo et al. (26), who demonstrated that none of the retreatment methodologies used were able to totally remove the residual filling intracanal material using micro-CT. High-resolution micro-CT investigations consistently demonstrate that no current nickel-titanium file system, whether reciprocating or continuous rotary, is capable of completely clearing calcium silicate-based sealers and gutta-percha from complex or oval canal spaces. Because bioceramic sealers establish a tight chemical and micromechanical bond with dentin, mechanical files primarily strip the core gutta-percha mass while leaving thin layers or islands of sealer in uninstrumented buccal and lingual recess extensions (6, 25, 26).

Recent investigations have evaluated adjunctive retreatment approaches, including ultrasonic activation and laser-assisted techniques, to improve the removal of root canal filling materials. Gupta et al. reported that advanced activation methods may enhance the disruption and removal of residual obturation remnants when compared with conventional instrumentation alone. Future studies combining reciprocating instrumentation with ultrasonic or laser-assisted activation may provide additional insight into optimizing retreatment outcomes in oval root canals (27).

From a clinical perspective, these experimental findings demonstrate that single-file reciprocating protocols matching the canal size (such as Reciproc R40) offer a time-efficient alternative for removing core obturation masses from oval canals compared to multi-file retreatment sequences with smaller terminal diameters. However, because mechanical instrumentation alone failed to achieve complete material clearance in oval configurations filled with bioceramic sealer, these *in vitro* observations suggest that mechanical preparation alone is insufficient. While adjunctive irrigation modalities, such as passive ultrasonic or acoustic activation, are frequently proposed in endodontic literature to disrupt remnants in uninstrumented anatomical recesses, future studies are required to directly evaluate their additive efficacy in this specific setting.

Finally, certain limitations must be considered when interpreting these findings. The use of clinical CBCT entails distinct imaging limitations that must be acknowledged. Clinical CBCT operates at a substantially lower spatial resolution (0.12 mm voxel size) compared to high-resolution Micro-CT. Consequently, CBCT cannot reliably detect tiny remnants or thin radiopaque films of material below its spatial resolution limit. Furthermore, due to gray-scale overlap and attenuation artifacts, standard CBCT cannot differentiate residual gutta-percha from CeraSeal sealer; thus, measurements reflect the combined total volume of residual obturation material. While quantitative volumetric assessment at clinical CBCT resolution provides valuable translational insights regarding macro-remnant removal, future high-resolution Micro-CT investigations are required to evaluate microscopic sealer-dentin interface clearance.

Additionally, a primary methodological consideration of this study lies in the structural, dimensional, and sequence difference between the two commercial file systems. While the RC R40 instrument matched the initial canal preparation diameter (size 40,.06 taper), the terminal instrument in the PR protocol (D3) features a significantly smaller tip diameter (size 20,.07 taper) and relies on a three-file sequence. Consequently, this investigation evaluated the holistic efficiency of two distinct, manufacturer-recommended clinical retreatment protocols rather than isolating kinematic movement as a single independent variable. The superior cleaning efficacy and procedural speed of the RC protocol represent the combined influence of matched apical sizing, cross-sectional chip space, sequence simplicity, and reciprocating movement, rather than the superiority of reciprocating kinematics in isolation.

Within the limitations of this *in vitro* study, neither the single-file RC protocol nor the multi-file PR protocol completely removed gutta-percha and CerSeal sealer from oval root canals. However, the RC protocol demonstrated significantly higher overall volumetric cleaning efficacy and required less active instrumentation time than the PR protocol. Because mechanical file systems leave residual filling mass in uninstrumented oval recess extensions, further experimental investigations are necessary to evaluate whether supplementary irrigation activation strategies can enhance material clearance.

## Data Availability

The raw data supporting the conclusions of this article will be made available by the authors, without undue reservation.
